# Endogenous CHRNA7-ligand SLURP1 as a potential tumor suppressor and anti-nicotinic factor in pancreatic cancer

**DOI:** 10.18632/oncotarget.24312

**Published:** 2018-01-24

**Authors:** Verena M. Throm, David Männle, Thomas Giese, Andrea S. Bauer, Matthias M. Gaida, Juergen Kopitz, Thomas Bruckner, Konstanze Plaschke, Svetlana P. Grekova, Klaus Felix, Thilo Hackert, Nathalia A. Giese, Oliver Strobel

**Affiliations:** ^1^ European Pancreas Centre/EPZ, Department of General, Visceral and Transplantation Surgery, University Hospital Heidelberg, Heidelberg, Germany; ^2^ Institute of Immunology, University Hospital Heidelberg, Heidelberg, Germany; ^3^ Department of Functional Genomics, DKFZ, Heidelberg, Germany; ^4^ Institute of Pathology, University Hospital Heidelberg, Heidelberg, Germany; ^5^ Institute of Medical Biometry and Informatics/IMBI, University Hospital Heidelberg, Heidelberg, Germany

**Keywords:** SLURP1, CHRNA7, pancreatic cancer, nicotine

## Abstract

Smoking is associated with increased risk and poorer prognosis of pancreatic ductal adenocarcinoma (PDAC). Nicotine acts through cholinergic nicotinic receptors, preferentially α7 (CHRNA7) that also binds the endogenous ligand SLURP1 (Secreted Ly-6/uPAR-Related Protein 1). The clinical significance of SLURP1 and its interaction with nicotine in PDAC are unclear. We detected similar levels of SLURP1 in sera from healthy donors and patients with chronic pancreatitis or PDAC; higher preoperative values were associated with significantly better survival in patients with resected tumors. Pancreatic tissue was not a source of circulating SLURP1 but contained diverse CHRNA7-expressing cells, preferentially epithelial and immune, whereas stromal stellate cells and a quarter of the tumor cells lacked CHRNA7. The CHRNA7 mRNA levels were decreased in PDAC, and CHRNA7^high^-PDAC patients lived longer. In CHRNA7^high^ COLO357 and PANC-1 cultures, opposing activities of SLURP1 (anti-malignant/CHRNA7-dependent) and nicotine (pro-malignant/CHRNA7-infidel) were exerted without reciprocally interfering with receptor binding or downstream signaling. These data suggested that the ligands act independently and abolish each other’s effects through a mechanism resembling functional antagonism. Thus, SLURP1 might represent an inborn anti-PDAC defense being sensitive to and counteracting nicotine. Boosting SLURP1-CHRNA7 interaction might represent a novel strategy for treatment in high-risk individuals, i.e., smokers with pancreatic cancer.

## INTRODUCTION

Smoking is the strongest non-genetic risk factor for pancreatic ductal adenocarcinoma (PDAC) and is associated with poor survival in PDAC patients [1]. Many compounds in cigarette smoke, including the nicotine-derived nitrosamines, nicotine-derived nitrosamine ketone (NNK), and N-nitrosonornicotine (NNN), have been shown to cause DNA damage, leading to the activation of oncogenes and the inactivation of tumor suppressors [2, 3]. Other compounds, such as nicotine, are not carcinogens *per se*, but do promote tumor progression. Clinical data have suggested that smoking promotes disease progression and worsens the outcome of patients with pancreatic cancer [4–7]. The preclinical PDAC models using transgenic Ela-KRAS and KPC mice have proven ability of nicotine to promote initiation and progression of pancreatic cancer [8]. Treatment with nicotine for 86 weeks accelerated tumor formation and promoted aggressiveness of established tumors by inducing epithelial-mesenchymal transition (EMT), increasing number of the circulating cancer cells and their dissemination to the liver. Fully transformed cells acquired gene expression patterns and functional characteristics of cancer stem cells. The ability of nicotine to increase the aggressiveness of PDAC cells is well studied *in vitro* and *in vivo* [9–13]. Nicotine may promote the proliferation, migration, invasion, stemness, and EMT in the pancreatic tumor cells directly [14–24], as well as through paracrine loops involving tumor-associated fibroblasts and HGF-MET signaling [6].

Nicotine may bind to multiple nicotinic acetylcholine receptors and activate different signaling pathways. The nicotinic cholinergic receptor α7 (α7 nAChR/NACHR7, encoded by CHRNA7 gene, and called CHRNA7 through the manuscript), a ligand-dependent Ca^2+^-channel, has been particularly linked to the progression of smoking-related cancers [8, 9, 12, 14, 25]. In contrast to other nicotinic acetylcholine receptors, CHRNA7 shows no desensitization but rather up-regulation upon chronic nicotine stimulation [26]. The CHRNA7-dependent effects of nicotine may be transduced by adrenaline [27], consequently activating β-adrenergic receptors and promoting the release of VEGF [28], EGF [29], and arachidonic acid [30]. Moreover, CHRNA7 signaling may directly activate PI3kinase, MAP-kinase, and NF-kB pathways, known promoters of tumor angiogenesis and proliferation [31]. Metastasis formation is also affected by nicotine/CHRNA7 interactions due to the activation of JAK2/STAT3 and MEK/ERK1/2 signaling and up-regulation of MUC4 [14].

In the course of binding to CHRNA7, exogenous noxious agents compete with the endogenous ligands. The CHRNA7 ligand SLURP1 (secreted Ly-6/uPAR related protein 1) is a member of the subfamily of snake venom neuro- and cardiotoxins lacking the glycosylphosphatidylinositol (GPI)-anchoring signal sequence. Present in blood and urine, this hormone-like protein is secreted by neurons, the aerodigestive and mucocutaneous epithelia, cornea, fibroblasts, lymphocytes, and dendritic cells [32–35]. Mutations in SLURP1 have been associated with Mal de Meleda, an inflammatory palmoplantar hyperkeratosis [36]. Studying this disease has established SLURP1 as a marker of epidermal differentiation [33] and as a factor suppressing the secretion of the inflammatory cytokines TNFα, IL1, IL-6, and IL-8 [37, 38]. Further studies have revealed oncosuppressive effects of SLURP1. Mutations of SLURP1 are associated with a higher risk of developing melanoma [39] and mucocutaneous carcinoma [40]. Furthermore, SLURP1 protects esophageal keratinocytes from malignant transformation mediated by exposure to nitrosamine, and its production in malignant keratinocytes is diminished [41, 42]. Oncosuppressive activity of SLURP1 was related to its ability to inhibit activation of the oncogenes (ETS1, NRAS, SRC, AKT1, KIT, and RB1), the cell cycle regulator CDKN2A, the transcription factor STAT3 and the anti-apoptotic mediator BCL2. In contrast, down-regulation of TNFα and the tumor suppressor SERPINB5 was blocked by SLURP1. Moreover, SLURP1 was shown to exert anti-proliferative effects in human oral keratinocytes and HT29 colorectal adenocarcinoma cells [43, 44].

Therefore, we sought to determine whether SLURP1 exerts tumor-suppressive activity in pancreatic cancer cells and, as an endogenous CHRNA7 ligand, is able to block the tumor-promoting effects of nicotine in PDAC.

## RESULTS

### High pancreatic expression of CHRNA7 is associated with better postoperative survival in PDAC patients

**Figure 1:**
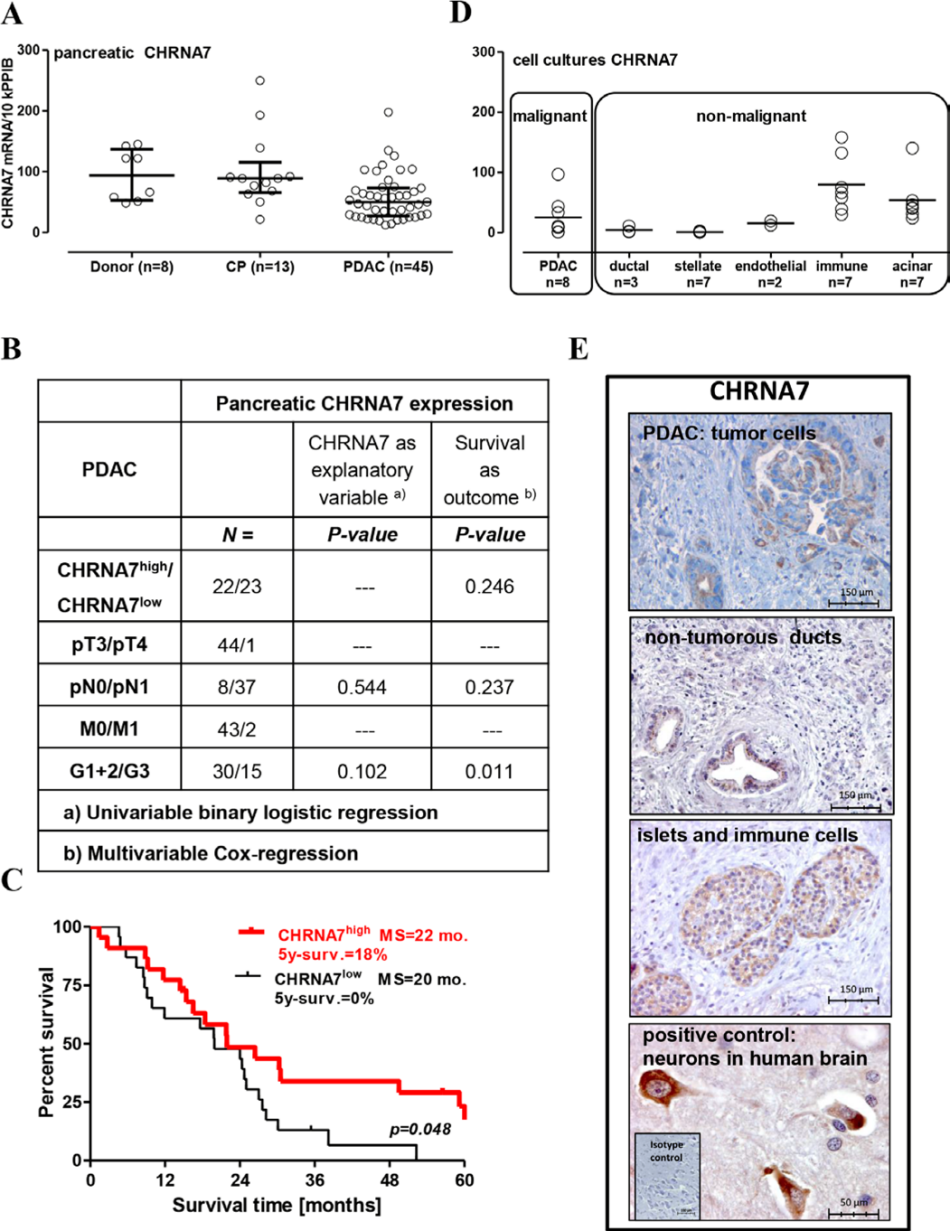
Preservation of CHRNA7 expression in the pancreas is associated with better prognosis for operable PDAC patients (**A**) CHRNA7 mRNA expression was determined by qRT-PCR in 66 pancreatic samples obtained from the organ donors and patients with chronic pancreatitis (CP) or pancreatic adenocarcinoma (PDAC). The groups were compared using the Kruskal-Wallis test (*p* = 0.004) with Dunn’s procedure, which established significant down-regulation of CHRNA7 in PDAC patients (*p* < 0.05). (**B**) Pancreatic CHRNA7 mRNA expression is not associated with tumor size, metastasis to lymph nodes, or differentiation grade. (**C**) Dividing the PDAC patients with resected tumors into CHRNA^high/low^ groups according to the mRNA level in the Kaplan-Meier survival analysis revealed longer survival in the CHRNA^high^ group (cut-off = 50 transcripts/10k PPIB; log-rank test *p* = 0.048), MS: median survival. (**D**) Specific contribution of the major cell types populating cancerous pancreatic lesion to overall CHRNA7 mRNA expression was evaluated by qRT-PCR analysis of the primary cell cultures (acinar, stellate/stromal, immune) and established cell lines (endothelial HUVEC/HMDEC, pancreatic ductal normal HPDE and tumor AsPC-1, BxPC-3, Capan-1, COLO357, MiaPaca2, PANC-1, SU.86.86, T3M4). (**E**) Immunohistochemical analysis located the CHRNA7 protein in pancreatic islets, immune cells, normal ducts, and cancer cells. Neuronal staining in human brain tissue served as a positive control.

Expression of the CHRNA7 receptor and its endogenous ligand SLURP1 in pancreatic specimens was analyzed using qRT-PCR and immunohistochemistry. By qRT-PCR analysis, we found that CHRNA7 mRNA was expressed in all analyzed samples: normal (donor), inflammatory (chronic pancreatitis/CP), and cancerous (PDAC) (Figure 1A). The median level of about 100 transcripts/10k PPIB (peptidylprolyl isomerase B, a house-keeping gene) was similar between normal and CP groups, but reduced by half in PDAC. CHRNA7 expression did not correlate with clinico-pathological parameters (Figure 1B) or smoking status (*p* = 0.239). Nevertheless, patients with higher CHRNA7 expression in resected pancreatic tumors had better survival compared to those with a lower level of expression (HR = 0.51, 95% CI = 0.26–0.99; 5-year survival 18% vs. 0%; Figure 1C).

PDAC is a complex tissue composed of various cell types. The epithelial cancer cells are embedded into a desmoplastic microenvironment composed by myofibroblasts (pancreatic stellate cells/PSC), hypertrophic vessels and nerves, infiltrating immune cells, and atrophic acini. qRT-PCR analysis of the primary and established cultures representing major cell types revealed that epithelial and immune cells, but not stromal PSC, expressed CHRNA7 mRNA (Figure 1D). The highest levels were measured in acinar and immune cells, and in 3 of 8 PDAC cell lines. Two PDAC cell lines lacked CHRNA7 mRNA, and three others contained only a few transcripts, comparable with normal ductal epithelium and endothelium. This pattern was in agreement with previously published findings [6, 15, 18, 21, 45].

Immunohistochemistry (IHC) confirmed the observed pattern, with anti-CHRNA7 antibodies staining malignant and also non-malignant epithelium (acini, islets, normal ducts), and immune cells but not stromal cells (Figure 1E). The staining intensity in the pancreas was rather weak compared to that of the neurons in the human brain used as a positive control. A granular cytoplasmic and apical membranous accumulation of CHRNA7 in tumor cells was observed in 25 out of 34 analyzed pancreatic specimens with a heterogeneous distribution pattern (10–40% of tumor cells), independent of the clinico-pathological parameters, including N (*p* = 0.672), M (*p* = 0.376), G (*p* = 0.971) status, smoking (*p* = 0.684) or survival (HR = 1.287; 95% CI = 0.58–2.83, *p* = 0.532).

Together, qRT-PCR and IHC analyses imply that the acini constitute the major source of the CHRNA7 expression in the normal pancreas. Progressive acinar loss and development of the CHRNA7-negative desmoplasia reduce the CHRNA7 expression level. Apparently, infiltrating immune cells might compensate for this drop much more efficiently than tumor cells, thus explaining the more robust maintenance of CHRNA7 expression in CP compared to PDAC tissues. Notably, CHRNA7-immunopositivity of the tumor cells not only lacked own prognostic relevance but also did not correlate with the total CHRNA7 mRNA content in the PDAC lesions (Rho = 0.091; *p* = 0.580). Whether the association of the CHRNA7 mRNA with the PDAC outcome is confounded by CHRNA7-positive non-malignant environment, remains to be determined.

**Figure 2:**
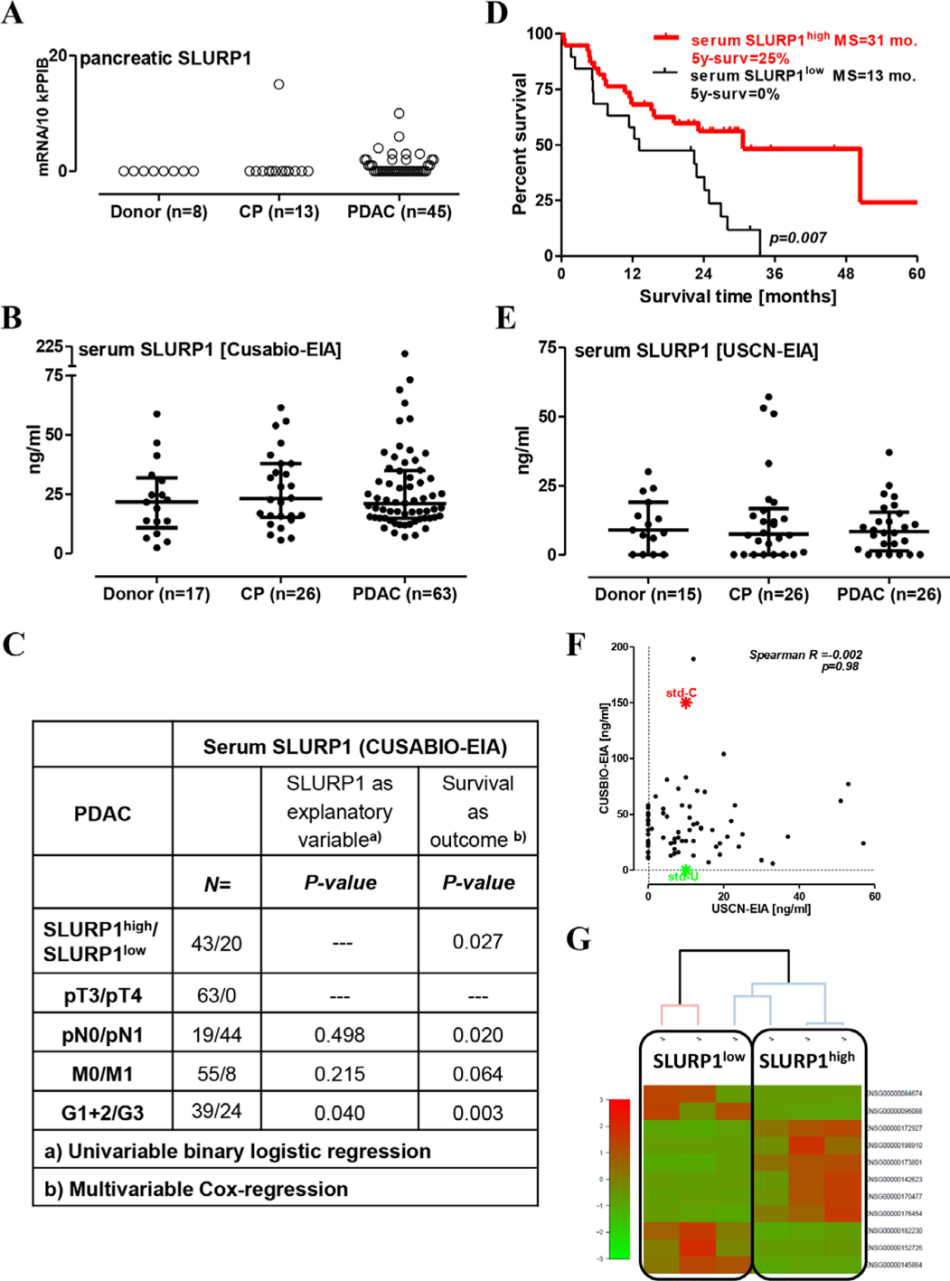
High level of circulating SLURP1 is associated with better survival in operable PDAC patients (**A**) The measurement of pancreatic SLURP1 mRNA expression with qRT-PCR in 66 pancreatic samples revealed the lack of expression in non-malignant pancreatic tissues and most PDAC lesions. (**B**) The concentration of SLURP1 was measured in 106 human serum samples using a commercial EIA kit produced by CUSABIO. Multiple comparison testing revealed a lack of difference between the analyzed groups (*p* = 0.643). (**C**) Circulating SLURP1 levels correlated with the grade of differentiation but not with any of the TNM parameters. (**D**) Dividing the PDAC patients with resected tumors into SLURP1^high/low^ groups according to the preoperative level of circulating SLURP1 for the Kaplan-Meier survival analysis revealed significantly longer survival of SLURP1^high^ patients (cut-off = 16 ng/ml; log-rank test *p* = 0.007), MS: median survival. (**E**) The concentration of SLURP1 was re-measured in 67 human serum samples using a commercial EIA kit produced by USCN and confirmed the lack of difference between the analyzed groups (*p* = 0.951). (**F**) Nevertheless, the USCN-EIA recognized the CUSABIO standard but not vice versa, and the USCN values for SLURP1 were three-fold lower and lacked any correlation with the CUSABIO values. Additional information is presented in Table 1. (**G**) Clustering analysis of the RNAseq data obtained for the PDAC specimens corresponding to the serum samples with low and high SLURP1 content.

In contrast to CHRNA7, SLURP1 was hardly detectable in pancreatic tissues (below the 15 copies/10k PPIB; Figure 2A). SLURP1 mRNA was absent in normal donor samples but present in a single CP probe and in a quarter of the cancerous lesions. The anti-SLURP1 antibody stained esophageal but not pancreatic tissue sections (data not shown), indicating that SLURP1 mRNA-expressing cells might be an extremely rare event in a fraction of pancreatic tumors.

### High preoperative serum levels of SLURP1 are associated with prolonged survival in patients with resected pancreatic tumors

Screening of the proprietary (ArrayExpress accession number E-MTAB-1791) or public (Oncomine, Gene Cards, Human Protein Atlas) expression databases confirmed the pancreatic SLURP1^−^/CHRNA7^+^ profile excluding pancreatic tissue as an ‘internal’ source of SLURP1 (data not shown). SLURP1, however, can be produced by different cell types, particularly those of mucocutaneous and immune origin, and has previously been found in biofluids such as urine and the hemofiltrate from patients with chronic renal disease [32–34]. We, therefore, hypothesized that SLURP1 of extrapancreatic origin may be released into the circulation to act through pancreatic CHRNA7 in an endocrine fashion.

To quantify SLURP1 protein levels in human sera, we used a commercially available ELISA kit manufactured by CUSABIO. The CUSABIO-EIA detected an average of 23 ± 4 ng/ml of SLURP1 in the sera of the healthy donors (median of 22 ng/ml with IQR = 2–32), and this level was not different in the CP and PDAC groups (Figure 2B). Dispersed within the logarithmic range, SLURP1 values did not associate with smoking status (*p* = 0.835) or advanced PDAC stages with metastasis to regional lymph nodes (N1) or distant organs (M1). A higher level of SLURP1 was associated with a better grade of histological differentiation, but by multivariable analysis emerged as a parameter independently associated with prolonged survival in the patients with resected pancreatic tumors (Figure 2C). SLURP1^high^-patients had a three-fold longer median survival time compared to SLURP1^low^ patients (HR for death = 0.36, 95% CI = 0.17–0.70; 5-year survival 25% vs. 0%; Figure 2D). Of note, combined sSLURP1^high^/pCHRNA7^high^-positivity tended to provide a modest prognostic benefit compared to other patients; it however did not reach the level of significance (log-rank test for trend *p* = 0.138), most probably due to the insufficient sample size for these subgroups.

To verify these findings, we used another commercial kit for SLURP1 manufactured by USCN/Cloud-Clone. Re-measurement of the selected serum samples using USCN-EIA confirmed the presence of SLURP1 in the bloodstream without significant changes in patients with pancreatic disorders (Figure 2E). However, the USCN values for SLURP1 were two to three times lower (average normal level of 11 ± 2 ng/ml; median of 9 ng/ml with IQR = 0–30) and did not correlate with the CUSABIO values (Figure 2F). USCN-EIA failed to detect SLURP1 in a quarter of the CUSABIO-positive sera, and this seronegativity was evenly distributed among donor (27%), CP (27%), and PDAC (23%) patients. Detailed analysis, however, showed that it affected one-half of the early-stage PDAC patients (pN0/UICC Stage IIa) but only a single late-stage PDAC patient with regional lymph node metastasis (pN1/UICC Stage IIb) (56% vs. 6%, respectively; Fisher χ^2^-test *p* =0.01; Table 1). Notably, USCN-EIA recognized only a 1/15^th^-fraction of the CUSABIO standard, which is of a eukaryotic origin; CUSABIO-EIA failed to recognize the prokaryotic USCN standard. We therefore speculated that CUSABIO-EIA and USCN-EIA recognize different forms of SLURP1, the balance of which might be affected by PDAC (see discussion).

**Table 1:** Measurement of SLURP1 in human serum by EIA

	CUSABIO-EIA	Cloud-Clone/USCN-EIA
	**General Information**	
**Immunogen‘s production**	CHO cell line (eukaryotic)	E. coli bacteria (prokaryotic)
**Immunogen/Standard**	full-length recombinant protein	full-length recombinant protein
**Posttranslational modifications (e.g., disulfide bonding)**	abundant	limited
**Coating antibody**	mouse monoclonal IgG	mouse monoclonal IgG
**Detecting antibody**	biotin-labeled mouse monoclonal IgG	biotin-labeled rabbit polyclonal IgG
**Detecting reagent/substrate**	avidin-HRPO/TMB	avidin-HRPO/TMB
**Standard detection range**	5–300 ng/ml	0.156-10 ng/ml
**Dilution of sera samples**	1:2	1:10
	**Measurements**	
**No. of patients:**	106	67
**Donor**	23 ± 4 ng/ml *n* = 17	11 ± 3 ng/ml *n* = 15
**CP**	27 ± 3 ng/ml *n* = 26	13 ± 3 ng/ml *n* = 26
**PDAC**	28 ± 3 ng/ml *n* = 63	10 ± 3 ng/ml *n* = 26
**Frequency of SLURP1- negative serum:**		
**Donor**	0%	27%
**CP**	0%	27%
**PDAC**	0%	23%
**PDAC- pN0**	0%	56%
**PDAC- pN1**	0%	6%
**Standards’ inter-EIA cross-reactivity:**		
** CUSABIO-Standard**	150 ng/ml	10 ng/ml
** USCN-Standard**	0 ng/ml	10 ng/ml

To substantiate our claim of SLURP1 having pathobiological relevance because of endocrine-like impact on CHRNA7-positive pancreatic lesions, we attempted to establish a connection between the level of SLURP1 in serum and the molecular landscape of pancreatic tumors. For that, we used whole-genome RNAseq data (collaborative project HIPO015 with Heidelberger Center for Personalized Oncology, DKFZ-HIPO, Heidelberg, Germany) for tissue specimens taken from the PDAC patients with the low (*n* = 3) and high (*n* = 3) serum SLURP1 values. Indeed, clustering analysis revealed distinct gene expression patterns in accordingly stratified PDAC tissues (Figure 2G), to be further explored in higher-sized cohorts.

### Anti-malignant and anti-nicotinic potential of SLURP1 in PDAC cells

In order to function as an inborn anti-PDAC mechanism, circulating SLURP1 should bind CHRNA7 on pancreatic tumor cells and exert an anti-tumor effect. To estimate the oncosuppressive potential of SLURP1, we analyzed the functional responses of PDAC cells to SLURP1 and nicotine *in vitro*.

**Figure 3:**
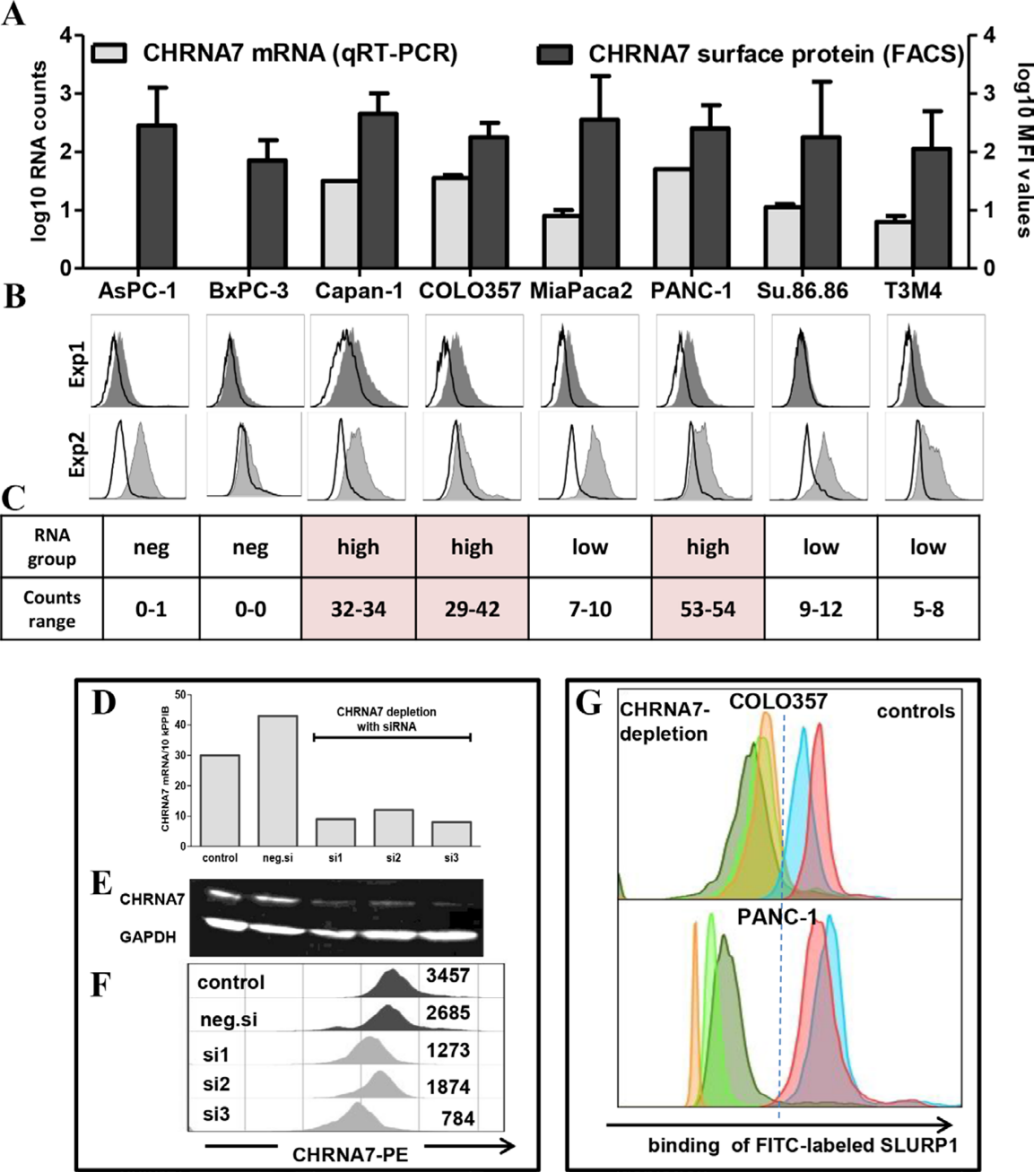
CHRNA7 is a major binding site for SLURP1 on PDAC cells (**A**) Simultaneous FACS and qRT-PCR analyses revealed CHRNA7-positivity of all eight tested PDAC cell lines, whereby membranous CHRNA7-staining showed significant inter-experimental variability and lack of correlation with RNA levels. The graph summarizes the data of two independent experiments shown below and measuring CHRNA7 mRNA (qRT-PCR; log10-transformed number of transcripts/10k PPIB) and surface protein expression (log10-transformed mean fluorescence intensity/MFI values) in unrelated passages of each cell line. (**B**) Individual FACS histograms illustrating intra- and inter-experimental variability are shown as an overlap of anti-CHRNA7-IgG (gray tinted area) and rabbit IgG (bold black line; isotype control) profiles; the difference in their geometric MFIs was used to calculate final MFI value for each culture. (**C**) qRT-PCR data demonstrating the inter-experimental stability of CHRNA7 mRNA expression also revealed the existence of the two groups, whereby CHRNA7^high^-group but not CHRNA7^neg/low^-group showed RNA-agreeable, stable pattern of protein expression. (**D**–**F**) The siRNA-based depletion of CHRNA7 confirmed the specificity of the reagents such as primers (qRT-PCR, D) and antibodies (Western blot, E, and FACS, F). As illustrated for COLO357 cultures, transfection with three commercially available CHRNA7-specific siRNA sets (si#1, si#2, si#3) eliminated over 75% of CHRNA7 mRNA and protein compared to the transfection of cells with negative control siRNA set (neg.si) or left untreated (control). (**G**) siRNA-based depletion of CHRNA7 indicated the preferential binding of SLURP1 to this receptor on PDAC cells. For comparison, FITC-conjugated SLURP1 was added to COLO357 and PANC-1 cells transfected with three different CHRNA7-siRNA sets (‘CHRNA7-depletion’ group depicted by grey-, green- and rose-colored profiles for si#1, si#2 and si#3, respectively) and to cells remaining intact or transfected with negative siRNA (‘controls’ group depicted by blue and lilac-colored profiles).

To this end, we first confirmed the absence of SLURP1 and the abundance of CHRNA7 in different PDAC cell lines. An analysis of eight PDAC cell lines by qRT-PCR and ELISA corroborated the SLURP1-negativity of PDAC tumor cells (data not shown). Simultaneous qRT-PCR and FACS analyses confirmed expression of CHRNA7 in tumor cells but also revealed certain discrepancies (Figure 3A–3C). A comparison of the data obtained for each cell line taken twice as independent passage showed a lack of correlation between RNA and surface protein expression (Exp1: *p* = 0.624, Exp2: *p* = 0.133). Furthermore, inter-experimental reproducibility was strong for RNA (Exp1 vs. Exp2: Pearson’s r = 0.971, *p* < 0.001) but not for MFI (mean fluorescent intensity Exp1 vs. Exp2: r = −0.084, *p* = 0.843) patterns. Notably, observed discrepancies represented a striking feature of the PCR^neg/low^-cells. In contrast, the PCR^high^-group repeatedly demonstrated a highly stable, concordant pattern. Consequently, PCR^high^ COLO357 and PANC-1 cells were chosen for the further studies.

The siRNA-based knockdown of CHRNA7 confirmed the specificity of the primers and antibodies employed for qRT-PCR, Western blot, and FACS analyses. Three different siRNA sets eliminated up to 75% of CHRNA7 RNA and protein in the COLO357 (Figure 3D–3F) and PANC-1 (data not shown) cultures. Importantly, FACS analysis demonstrated the specificity of SLURP1 binding CHRNA7 on the surface of the PDAC cells. As shown in Figure 3G, PDAC cells bound FITC-conjugated SLURP1, and this binding was greatly reduced in CHRNA7-depleted cultures by 85% in COLO357 and by 95% in PANC-1 cells.

**Figure 4:**
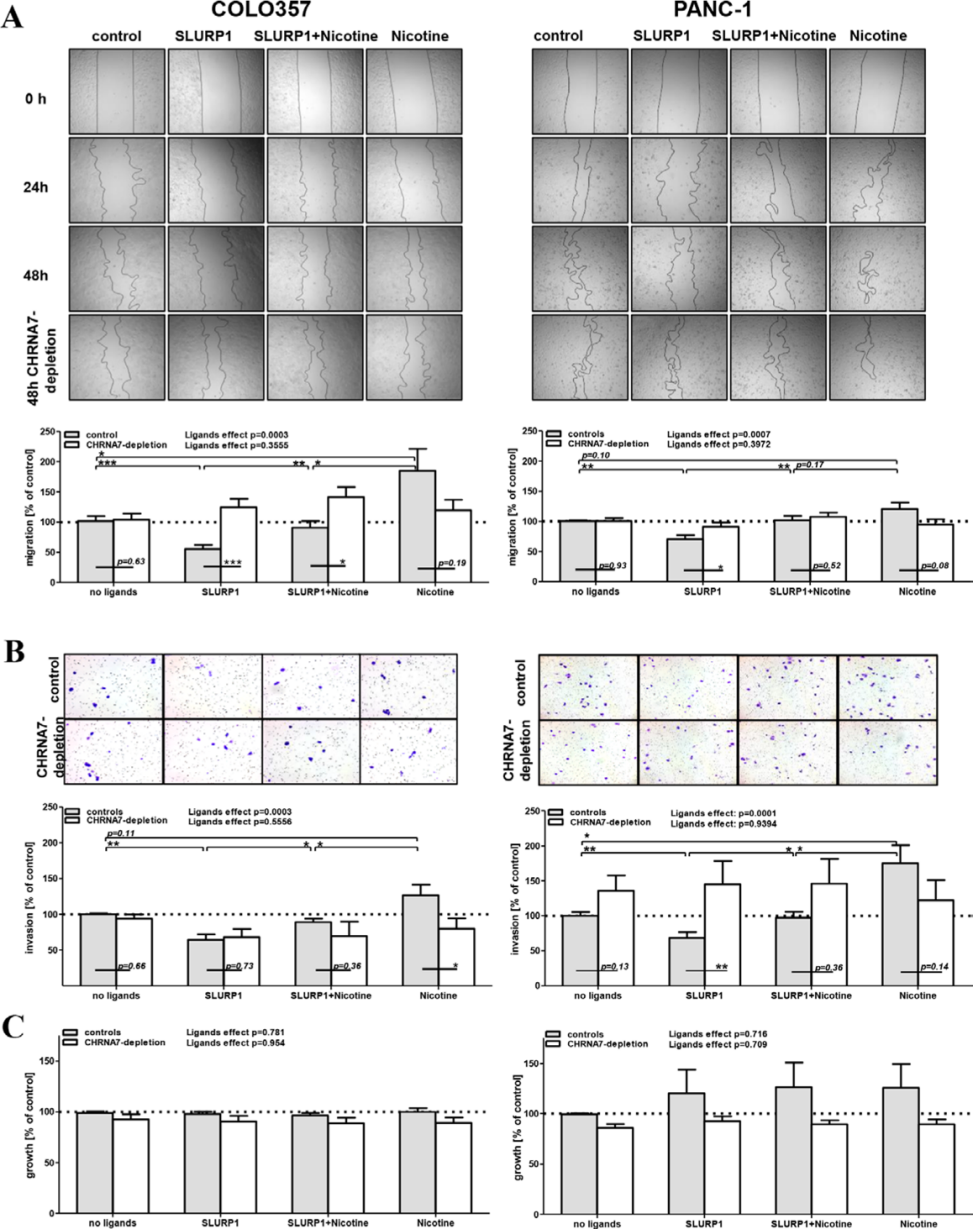
In PDAC cells, anti-malignant SLURP1 and pro-malignant nicotine are mutually opposing forces (**A**) The wound healing (scratch) assay, (**B**) The Matrigel-based invasion assay and (**C**) MTT-based growth assay revealed that without affecting proliferation of the PDAC cells, SLURP1 significantly reduced their migration and invasion (*p* < 0.01), whereas nicotine tended to promote these (*p* = 0.05–0.11). Co-application abolished these opposite effects (*p* < 0.05). CHRNA7-depletion abolished the action of the ligands (*p*-values < 0.0003 in controls vs. *p*-values > 0.36 upon CHRNA7-depletion), whereby this effect was evident for SLURP1, it was much weaker for nicotine. Upper panels: representative images from one experiment; lower panels: quantification of data obtained from four to eight independent experiments with control cultures (grey bars: pooled samples which have been left intact or transfected with negative control siRNA) and CHRNA7-depleted cultures (white bars: pooled samples which have been separately transfected with siRNA sets si#1, si#2 and si#3). The effect observed in the non-treated/non-transfected culture was set as control = 100% and served as a reference for normalization of the raw data in each experiment. The detailed description of the data processing and the exact numbers of the values per analyzed group are given in the Materials and Methods. The overall impact of the ligands treatment was determined with one-way ANOVA test separately for control and CHRNA7-depleted cohorts. Two-groups comparisons estimating effects of ligands or knockdowns have been performed with Welch’s *t*-test. Significant level shown as ^*^ = *p* < 0.05; ^**^ = *p* < 0.01; ^***^ = *p* < 0.001.

To test the anti-malignant and anti-nicotinic potential of SLURP1, we treated COLO357 and PANC-1 cells with SLURP1 (25 nM) and nicotine (100 nM), alone or in combination, and monitored their functional activity up to 72 h post ligand application. The wound healing (scratch) assay, Matrigel-based invasion analysis, and the MTT-based growth test showed that SLURP1 significantly inhibited migration and invasion by 25–50% (*p*-values < 0.01, Figure 4A–4B), whereas nicotine tended to promote it (*p*-values < 0.05–0.11); both without affecting proliferation (Figure 4C). Importantly, SLURP1 could reverse the pro-malignant activities of nicotine, whereby vice versa its anti-malignant activity was also attenuated by nicotine. Thus, co-application diminished the opposing actions of both ligands and yielded an “average” effect, returning cells to the basal level of activity.

Depletion of CHRNA7 by siRNA abolished the strong overall effect of the ligands on PDAC cells (*p*-values > 0.36 vs. *p*-values < 0.007, respectively, as shown at the top of each of the four assays summarized in Figure 4A–4B). The analyzed cultures differed in their sensitivity to knockdowns: the CHRNA7-depletion neutralized SLURP1 activity more effectively than that of nicotine (Figure 4A–4B depicts *p*-values for all 16 compared ‘control-depletion’ pairs). Such a pattern is in line with previous observations, suggesting the singularity of CHRNA7 as a binding partner for SLURP1 and CHRNA7-infidelity of nicotine, which is capable of binding other nicotinic receptors [9, 12, 18, 43, 46].

### Nicotine-independent binding and signaling of SLURP1 in PDAC cells

**Figure 5:**
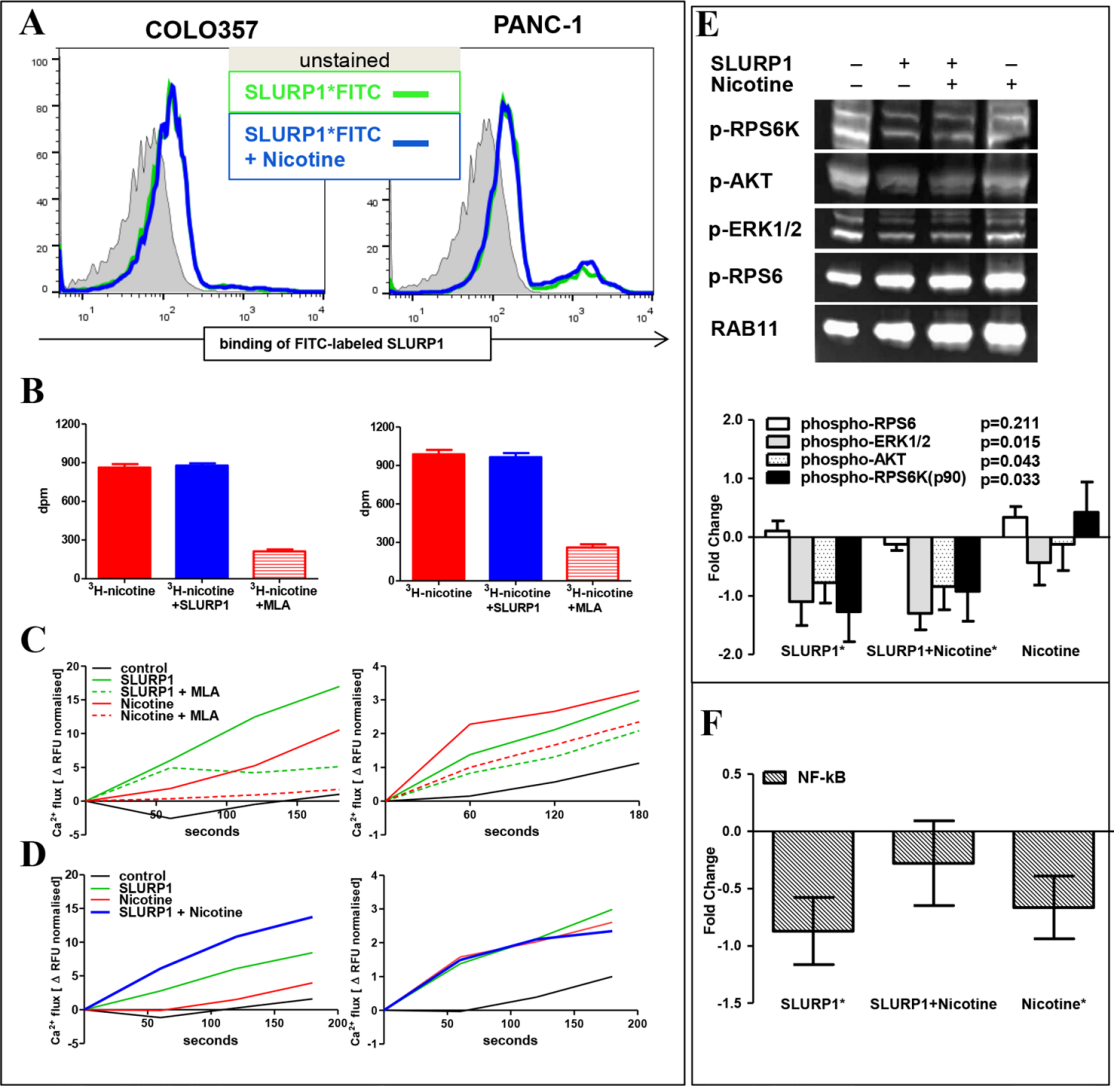
In PDAC cells, SLURP1 and nicotine occupy different binding sites and signal independently (**A**) FACS analysis of the PDAC cells showed no difference in binding of FITC-labeled SLURP1 in the absence (green line) or presence (blue line) of nicotine. (**B**) Radioligand assay showed no difference in binding of ^3^H-labeled nicotine in the absence (red bars) or presence (blue bars) of SLURP1, whereas exposure to a competitive CHRNA7-antagonist methyllycaconitine citrate (MLA) eliminated 80% of the binding. *dpm*: disintegrations per minute. (**C**) Both, SLURP1 and nicotine, induced MLA-sensitive Ca^2+^ influxes in COLO357 and PANC-1 cells. (**D**) Concurrent addition of SLURP1 and nicotine augmented Ca^2+^ influx in COLO357 (*p* = 0.01) and did no alter it in PANC-1 cultures. (**E**) Western blot analysis of the cellular lysates using the PathScan antibody cocktail showed that SLURP1 significantly inhibited basal phosphorylation of pAKT, pERK1/2, and p90RSK, but not of pRPS6 in COLO357, and this deactivation of potentially oncogenic signaling was maintained with concurrent exposure to nicotine. Nicotine alone showed no significant impact on the phosphorylation of these molecules. The lower panel summarizes data from four independent experiments and presents the quantification of the Western blot bands normalized to the RAB11 signal expressed as fold change over the untreated control. (**F**) TransAM-EIA assay, measuring the concentration of nuclear NF-κB in COLO357 cells, revealed that SLURP1 and nicotine significantly reduced the nuclear translocation of NF-κB when delivered alone but not together. The graphs summarize data from 5 to 14 independent experiments in duplicate or quadruplicate for each treatment. Significance level ^*^ = *p* < 0.05.

The most obvious explanation for the mutually weakened effects upon concurrent exposure to both ligands would be competitive binding of SLURP1 and nicotine to CHRNA7. However, the SLURP1^FITC^-binding assay performed in the presence of nicotine showed an intact fluorescence profile in PDAC cells (Figure 5A). In turn, a radio-ligand assay demonstrated that also SLURP1 cannot preclude ^3^H-nicotine binding to PDAC cells, whereas specific competitive CHRNA7-antagonist methyllycaconitine (MLA) greatly reduced it (Figure 5B). Such pattern suggests that, most probably, SLURP1 and nicotine do not compete for binding sites.

Since co-application did not appear to impede binding of SLURP1 or nicotine to PDAC cells, we analyzed signaling events downstream of CHRNA7, which is a ligand-dependent Ca^2+^-specific ion channel. Within seconds, SLURP1 triggered Ca^2+^ influx which showed strong sensitivity to MLA: down by 70% in COLO357 and by 30% in PANC-1 (Figure 5B–5C). Within minutes, the SLUPR1 signal was transduced to the AKT/ERK pathway and greatly reduced the phosphorylation of pAKT, p90RSK, and pERK1/2 proteins but not that of the mTOR downstream target, pRPS6 (Figure 5D). SLURP1 and nicotine blocked the translocation of NF-kB to the nucleus (Figure 5E). Notably, nicotine also triggered Ca^2+^ influx into PDAC cells, which was further enhanced in the presence of SLURP1 in COLO357 and preserved in PANC-1 cells (Figure 5C). However, nicotine did not affect AKT/ERK pathways, either basal or SLURP1-inhibited.

Taken together, our functional and molecular studies show that opposing activities of SLURP1 (anti-malignant/CHRNA7-dependent) and nicotine (pro-malignant/CHRNA7-infidel) were exerted without reciprocally interfering with receptor binding or downstream signaling. These data suggested that the ligands act independently and abolish each other’s effects through a mechanism resembling functional antagonism (see discussion).

## DISCUSSION

Although smoking is an important non-genetic risk factor and tumor promoter for PDAC, the underlying pathobiology is poorly understood. Our study shows that the protein SLURP1, an endogenous ligand for the nicotinic CHRNA7, has strong anti-PDAC potential, and can be found in the bloodstream at a level that might be sufficient to weaken the pro-malignant activities of nicotine. At the same time, nicotine attenuates the anti-malignant activities of SLURP1 suggesting that smoking could weaken the natural anti-tumor defense provided by SLURP1.

The spectrum and magnitude of SLURP1-mediated oncosuppression in PDAC cells are in partial agreement with previously published findings. Firstly, SLURP1 reduced the migration and invasion of PDAC cells. Whereas the anti-migratory effect of SLURP1 in PDAC cultures was comparable to that in keratinocytes [47], the anti-proliferative effect shown for colon cancer cells and keratinocytes [43, 44] was missing. The anti-invasive effect of SLURP1 was a novel finding. Secondly, SLURP1 weakened the nicotine-mediated migration and invasion while forfeiting its own anti-tumor potential. The mutual inhibition of SLURP1 and nicotine might occur through various mechanisms, for example, competition between SLURP1 and nicotine for binding sites, as it has been shown in keratinocytes using a radioligand assay [35]. On the other hand, SLURP1 has been described as an positive allosteric modulator, which potentiates the effect of acetylcholine but lacks an effect in the absence of acetylcholine [47, 48] – in contrast to non-competitive inhibition of the acetylcholine described by Lyukmanova et al. [43].These differing activities have been linked to ability of SLURP1 to engage ionotropic as well as metabotropic signaling through CHRNA7 [43, 49]. According to our data, SLURP1 induced Ca^2+^ influx, diminished the phosphorylation of pAKT/pERK but not pRPS6 (an mTOR target), and blocked nuclear NF-kB translocation in PDAC cells. This finding is in contrast to the results of Chernyavsky et al., who showed that SLURP1 upregulates ERK1 and NF-kB expression [49]. This deviation might be due to the different cell types used in these studies, i.e., esophageal epithelial cells versus pancreatic cancer cells. Numerous other reports have shown an antiinflammatory effect of SLURP1, supporting the down-regulation of NF-kB by SLURP1 [37, 38, 48]. NF-kB is also known for its tumor-promoting activities, as it promotes proliferation [50, 51], reduces apoptosis [52], and facilitates metastasis by down-regulating cellular adhesion molecules [51]. The reduction in NF-kB signaling by SLURP1 matches our *in vitro* and clinical observations revealing the anti-malignant potential of SLURP1. Concerning nicotine, we demonstrated that it did not affect SLURP1-triggered AKT/ERK/mTOR pathways but, surprisingly, reduced NF-kB translocation into the nucleus. This differs from studies that have shown the activation of NF-kB in multiple cell lines [27, 53], in line with the known proliferative and tumorigenic effect of nicotine. However, there are also studies showing inhibition [54] or a lack of effect towards NF-kB signaling [55]. Notably, the NF-kB inhibitory effect of nicotine was similar to that of SLURP1; simultaneous application of ligands only slightly weakened it.

Altogether, the spectrum of previously described SLURP1 activities ranges from agonistic to antagonistic and assumes a capability to trigger CHRNA7-coupled ionotropic as well as metabotropic signaling pathways [35, 43, 48, 49, 56]. Our study demonstrates that the action of SLURP1 and nicotine in PDAC cells is characterized by a non-competitive binding, sovereign signaling, and opposing activities of differing CHRNA7-dependency. This pattern suggests that these ligands act independently and interconnect through a mechanism most closely resembling functional antagonism – “a situation in which two agonists interact with different receptors and produce opposing effects” [57]; not to be confused with a chemical antagonism. Both, strong CHRNA7-dependency of SLURP1 and CHRNA7-infidelity of nicotine, are in line with previously published publications. An affinity purification assay excluded SLURP1 binding to α3, α4, α5, α6, β2, or β4 subunits and postulated CHRNA7 as a singular SLUPR1-receptor [43]. In contrast, nicotinic acetylcholine receptor subunits other than α7 have been well documented to bind nicotine. Furthermore, nicotine may permeate cellular membrane and activate mitochondrial CHRNA7s [9, 12, 18, 46, 58]. In this context, it should be noted that CHRNA7 is located on chromosome 15q11–14, a region known for a common inversion polymorphism, microdeletions, and segmental duplications [59]. These events distort the structure of CHRNA7 and are associated with Prader-Willi/Angelman syndrome and mental retardation [60]. Human-specific duplicons of CHRNA7 are encoded by the CHRFAM7A gene, consisting of the inverted CHRNA7 exons 5–10 fused with sequences of FAM7A [59]. Both receptors can bind SLURP1 and nicotine, but the downstream consequences may differ. For example, CHRFAM7A has been shown to reduce nicotine signaling via CHNRA7 [61]. In our study, we used three siRNA sets targeting CHRNA7, of which si#1 and si#2 targeted exclusively CHRNA7 only and si#3 targeted both CHRNA7 and CHRFAM7A. All depleting approaches similarly diminished the level of CHRNA7 and binding of FITC-labeled SLURP1, and restored SLURP1-inhibited migration, whereas invasion was reinstated in PANC-1 but not in COLO357 cultures. Whether SLURP1 and nicotine engage separate receptors or different CHRNA7-binding sites with inotropic/metabotropic bias and whether SLURP1 functions as an inverse agonist or ago-allosteric modulator remains to be determined.

Analysis of the clinical specimens established an association between postoperative survival and the levels of pancreatic CHRNA7 or preoperative systemic SLURP1 expression. It should be noted that the analyzed specimens came from patients with resected tumors [62]. Results of *in vitro* studies implied that SLURP1^high^-patients might be better protected against PDAC recurrence or progression through direct anti-malignant effects of SLURP1 on CHRNA7-positive tumor cells. Thus, further studies should establish the prognostic role of SLURP1 in inoperable PDAC patients, and also explore the possibility of its therapeutic application. Most probably, the direct anti-malignant effect of SLURP1 on PDAC cells is not the only mechanism behind SLURP1-driven oncosuppression: tumor cells were not a sole source of the pancreatic CHRNA7 expression in cancerous lesions. Various non-malignant ‘normal’ cells also expressed CHRNA7, thus providing a broad spectrum of targets for potential oncosuppression [8, 37, 63]. Distinct compartmentalization (CHRNA7-negative stroma vs. highly positive acinar and immune cells) has further hampered prognostic assessment: the significance of CHRNA7 might be confounded by the frequencies of cells having their own clinical relevance [64, 65]. An exact interpretation of the clinico-molecular associations requires an extensive quantification of the CHRNA7- and CHRFAM7A-positive cell types, which can be done only with prior clarification of the mechanisms responsible for RNA/surface protein discrepancy detected by our PCR/FACS measurements. The issues of alternative splicing, stability, and turnover should be taken in account while designing further studies [66–69].

The possible variability of SLURP1 molecules adds another layer of complexity. We used two different EIAs to quantify SLURP1 in human serum samples, whereby both kits employed a full-length recombinant protein as an immunogen to produce the antibodies. However, one was a eukaryotic system (Chinese hamster ovary/CHO cells, CUSABIO-EIA) while the other was a prokaryotic system (*E. coli* bacteria, USCN-EIA). The immunogens and the standards thus have undergone different post-translational modifications, particularly such structural changes as disulfide bonding. That processing greatly affects folding, tertiary structure and stability of SLURP1, a disulfide-bonded protein containing 10 cysteine residues forming five -S-S- bridges [32, 70]. Both EIAs revealed that the amount of SLURP1 in the circulation was similar in healthy, CP, and PDAC individuals. However, we observed a lack of correlation between individual EIA measurements, and the CUSABIO-EIA provided three-fold higher values without recognition of the USCN-standard, while USCN-EIA detected both standards but measured lower SLURP1 levels in serum samples. This pattern suggests that eukaryotic cells may produce SLURP1 molecules varying in the number or location of disulfide bridges. Their balanced production may be disturbed in aerodigestive and mucocutaneous epithelium reacting to an altered spectrum of circulating cytokines in inflammation and cancer [34, 71, 72]. Further studies are required to confirm assumed conformational modifications and to determine how they may affect SLURP1’s folding/stability and functioning.

In summary, we found that pancreatic epithelial cells, both normal and malignant, express the CHRNA7 receptor in the absence of local production of SLURP1, which, however, could be secreted at distant locations and delivered to the pancreas via the bloodstream. SLURP1 exerts direct anti-PDAC activity by controlling AKT, ERK and NF-kB signaling, and may oppose pro-malignant actions of nicotine through mechanisms that are not yet fully understood. We conclude that endogenous SLURP1 may suppress PDAC recurrence and/or progression and also protect against environmental risk factors such as smoking. However, smoking may compromise this inborn anti-PDAC defense. Therefore, the SLURP1-CHRNA7 interaction might represent a novel therapeutic target and should be further explored for PDAC prevention and therapy in high-risk individuals such as smokers.

## MATERIALS AND METHODS

### Clinical specimens

Serum and pancreatic tissue samples were obtained from the Pancobank of the European Pancreas Centre (EPZ/Department of Surgery, University Hospital Heidelberg; Ethical Approval Votes no. 301/2001 and 159/2002), a member of BMBH/Biomaterial Bank Heidelberg. Exact numbers of the specimens in the donor, chronic pancreatitis (CP) and pancreatic adenocarcinoma (PDAC) cohorts are presented in the Table 1, text and figures. The clinico-pathological parameters included age, gender, TNM classification, grade of tumor cell differentiation (G), smoking status, and postoperative overall survival time. All analyzed samples are derived from patients with operable pancreatic tumors; potentially curative resections were performed at the UICC stages IIA (25%), IIB (66%) or IV (9%) [62].

### Quantitative RT-PCR (qRT-PCR)

qRT-PCR was used to quantify SLURP1 and CHRNA7 mRNA expression in pancreatic tissues (8 specimen from organ donors, 13 CP specimen, and 45 PDAC specimen), in eight PDAC cell lines, and in the cultures of normal cells representing the major cell types in pancreatic lesions. The primary isolates have been done in our laboratory using pancreatic tissues (acinar and stromal pancreatic stellate cells/PSC preparations) and blood (immune preparations) as described previously [72–76]. Other cells have been purchased (endothelial HUVEC/HMDEC) or obtained as a kind gift (normal pancreatic immortalized cells HPDE6-E6E7-c7 cells; Dr. Ming-Sound Tsao). Esophageal RNA was purchased from BioCat (Heidelberg, Germany) and was used as a positive control for SLURP1 expression. All instruments and reagents for qRT-PCR analysis were purchased from Roche Applied Biosciences AG (Mannheim, Germany) and applied as described previously [77, 78]. In brief, mRNA extraction was performed with the MagNA Pure^®^ System; cDNA was synthesized with the First Strand cDNA synthesis kit and PCR analysis was performed using the LightCycler^®^480. Primers were obtained from Search-LC (Heidelberg, Germany). The SLURP1 and CHRNA7 copy numbers were normalized to those of the housekeeping gene peptidylprolyl isomerase B (PPIB) and are expressed as the number of specific transcripts per 10,000 PPIB copies (10k PPIB). CHRNA7 primers span nucleotides 1571–1772 in the exon 10–3’UTR encoding canonical C-term amino acids 491–502 present in alternatively spliced isoforms P36544–1 and P36544–2 but not in P36544–3.

### Immunohistochemistry

To localize SLURP1 and CHRNA7 protein in tissues, we stained formalin-fixed paraffin-embedded (FFPE)-sections with the respective antibodies using a standard immunohistochemistry protocol [65, 78]. For antigen retrieval, 4 μm-thick sections were heated in citrate buffer (pH 6) for 30 min at 96°C, and then blocked with methanol containing 3% H_2_O_2_ and a universal blocking reagent (BioGenex, San Ramon, USA). Exposure to primary antibodies was performed at 4°C overnight using rat anti-chicken/human CHRNA7 IgG (aa 365–384; Cat# ab24644; Abcam, Cambridge, UK), rabbit anti-human CHRNA7 IgG (aa31-42; Cat# ANC-007; Alomone, Jerusalem, Israel), or sheep anti-human SLURP1 IgG (aa22-103; cat# AF4479; R&D Systems, Minneapolis, USA). Control sections were incubated with respective isotype controls, i.e. rat, rabbit or sheep IgG. After washing in TBS with 0.05% Tween-20, slides were exposed for 45 min to horseradish peroxidase-labeled anti-rat (Amersham/GE Healthcare Europe GmbH, Freiburg, Germany) or anti-sheep (Santa Cruz Biotechnologies GmbH, Heidelberg, Germany) secondary antibodies, then incubated for 1 hour with DAB reagent (Dako, Hamburg, Germany) and counterstained with hematoxylin. The images were recorded using a light microscope and analyzed by AxioVision software (Zeiss, Oberkochen, Germany). Positive control sections of human brain and esophagus were provided by NCT-Biobank/BMBH (Institute of Pathology, Heidelberg, Germany).

### Enzyme-linked immunosorbent assay (EIA)

Biochemical methods estimated the blood concentration of SLURP1 at the 10 nM level (ca. 88 ng/ml) [32]. The single EIA-based study reported 13±5 ng/ml of SLURP1 in human plasma using urinary protein as an immunogen to produce the detecting antibodies [79]. To determine the exact physiological concentrations of SLURP1 in blood, we used commercially available EIA kits manufactured by CUSABIO Biotech Co., Ltd. (Wuhan, P.R. China; Cat# CSB-EL021784HU) and Cloud-Clone/USCN (Houston, USA; Cat# SEM240Hu). The human sera were first analyzed by the CUSABIO kit (*n* = 106). The samples were assayed at least twice in an independent manner, and all measurements underwent global normalization to reduce the impact of inter-lot variability. A subset of the samples analyzed with the CUSABIO kit (*n* = 67) was re-analyzed using the EIA from USCN. The studied PDAC cohorts and the EIA kits are described in Table 1. The major difference between the kits was the cellular source of the recombinant immunogen/standard: eukaryotic in the CUSABIO EIA and prokaryotic in the USCN EIA. Cross-reactivity testing showed that the USCN EIA recognizes the CUSABIO standard but the CUSABIO EIA does not recognize the USCN standard.

### Cell culture and treatments

The pancreatic cancer cell lines COLO357 and PANC-1 were cultured in RPMI 1640-medium supplemented with 10% FBS and 1% penicillin/streptomycin. For treatment, we used recombinant SLURP1 at 25 nM (Abnova, Taipei City, Taiwan) and nicotine at 100 nM (Tocris, Ellisville, USA; the dose corresponds to the level found in the serum of smokers [80]). The specific CHRNA7 antagonist methyllycaconitine (MLA) was used at 25 nM (Sigma-Aldrich, St. Louis, USA).

CHRNA7-depleted clones were generated using siRNA sets (Ambion #16708, #4392420 and #4392420, Huntington, UK) and HiPerFect transfection reagent (QIAGEN, Venlo, Netherlands) and compared to non-transfected cells or clones transfected with negative control siRNA (Ambion #4390843). The efficacy of siRNA-based CHRNA7 knockdown was evaluated using qRT-PCR, Western blot and FACS analyses.

### Radio-ligand assay

We used the radio-ligand assay to analyze binding of the nicotine to receptors on PDAC cells [81]. COLO357 and PANC-1 (10^6^ per well; 6-well plates) were washed with serum-free medium and exposed for 15 min at 4°C to 1 ml of 20 nM L-[N-methyl-^3^H]-nicotine (American Radiolabeled Chemicals/ARC Inc, St. Louis, MO, USA; specific activity 80 Ci/mmol), with or without 25 nM SLURP1 or 200 nM MLA. After three washing steps, the cells were lysed with 1 ml 0.5M NaOH, mixed with UltimaGold™ scintillation cocktail, and counted for radioactivity in a TRICARB 2900TR scintillation counter. The external standard method using tSIE as quench indicator was applied for dpm (disintegrations per minute) calculation. Untreated cultures produced mean dpm value of 1000. It corresponds to 5.63 fmol of bound [^3^H]-nicotine and, consequently, 3378 nicotine-binding sites per cell; 75% of that - MLA-sensitive ones (app.2500 per cell, comparable with other cell types, e.g. monocytic THP-1 [82]). The Receptor to Ligand ratio for nicotine (20–100 nM) and SLURP1 (25 nM) in the *in vitro* studies was estimated to exceed 1:5000.

### Flow cytometry (FACS)

Fluorescence activated cell sorting (FACS) analysis was used to determine the expression of CHRNA7 on the surface of tumor cells and to detect the binding of FITC-labeled SLURP1 to this receptor. For CHRNA7 detection, 10^6^ cells were blocked with Fc-blocking reagent (Miltenyi Biotec GmbH, Bergisch Gladbach, Germany) and stained with rabbit anti-human CHRNA7 antibody (which recognizes extracellular N-term in all three P36544 isoforms (Alomone Cat#ANC-007; aa31-42 in canonical sequence P36544-1)) for 35 min followed by a PE-labeled goat anti-rabbit antibody for 20 min. Control samples were left unstained or stained with rabbit IgG instead of the primary antibody (isotype control). For the binding assay, COLO357 and PANC-1 cells were incubated with recombinant SLURP1, which had been conjugated with FITC (SureLINK^TM^ Fluorescein (FITC) labeling kit; KPL, Gaithersburg, MD, USA), in combination with or without nicotine (in an equimolar or two times the concentration of SLURP1-FITC). To confirm the specificity of binding, the assay was performed with untreated and CHRNA7-depleted cells by siRNA. Fluorescence was measured with a flow cytometer (BD FACS Canto^TM^ II, Heidelberg, Germany) and analyzed using FlowJo® v10 software (Ashland, OR, USA).

### Western blot analyses

Western blot analyses were used to confirm the efficacy of CHRNA7-siRNA knockdown (72 h post-transfection) and to check SLURP1 ± nicotine-induced activation of AKT, ERK, and mTOR signaling. For the latter, 2.5 **×** 10^5^ PDAC cells were grown in serum-free medium in 6-well plates overnight and treated for 15 min with SLURP1, nicotine, a combination of both, or were left untreated. The lysates were prepared using SDS lysis and electrophoresis buffer containing protease and phosphatase inhibitors (Roche, Mannheim, Germany; Sigma-Aldrich), homogenized by ultrasound, separated on 4–12% Bis-Tris gel, and transferred to a nitrocellulose membrane. For immunodetection of the target proteins, we used a rabbit anti-CHRNA7 antibody (Alomone) or PathScan® Multiplex Western Cocktail I kit (Cell Signaling Technology, Danvers, MA, USA), followed by horseradish peroxidase-labeled anti-rabbit IgG (SCBT) and SuperSignal West Dura Extended Duration Substrate Kit (Pierce, Waltham, USA). The signals were recorded using a FUSION image acquisition system (Vilber Lourmat, Marne-la-Vallée, France). Band intensity was quantified using ImageJ software and normalized to the GAPDH or RAB11 levels, respectively.

### Wound healing (scratch) assay

The *in vitro* wound healing (scratch) assay was performed as described elsewhere [83] with non-transfected and siRNA-transfected PDAC cells. In brief, duplicate cultures of 5 × 10^5^ COLO357 and PANC-1 cells (*n* = 7 and *n* = 5 independent experiments, respectively) were plated in a 24-well plate, grown to 90% confluence, and scratched with a sterile 200 μl pipette tip. Then, the cells were washed with PBS and treated with SLURP1, nicotine or a combination of both for 48 h. Pictures were taken after 0, 24, and 48 h at 40 × magnification. The center of the plate was marked as a central reference point to ensure recording of the same area during the time course. Digital images were taken, and the scratch area was quantified using ImageJ software. The remaining cell-free area was measured as a percentage of the original scratch area at 0 h, and scratch closure was calculated as 100% minus remaining free area. In each experiment, the duplicate measurements have been averaged; the non-treated/non-transfected sample served as the 100%-reference level to express other values as a percentage of the control. For statistical analysis and graphical presentation, the obtained values have been separated according to the CHRNA7-status into control (non-transfected and negative control siRNA-transfected samples) and CHRNA7-depletion (si#1, si#2, and si#3- transfected samples) cohorts. The experiment was independently reproduced seven times for COLO357 (producing up to 14 control vs. 21 CHRNA7-depleted values) and five times for PANC-1 cells (10 vs. 15 values) for each ligand-treated group. Figure 4 depicts control groups as grey bars and the CHRNA7-depleted groups as white bars. The overall effect of the ligands (multiple comparisons) was evaluated using a one-way ANOVA test. The significance of the changes was calculated separately for basal and CHRNA7-depleted conditions, as indicated by *p*-values for ‘Ligands effect’ placed above the graph. The significant differences between any two analyzed groups have been calculated with Welch’s *t*-test and presented by the lines and stars, as indicated in the figure’s legend.

### Invasion assay

Non-transfected and siRNA-transfected PDAC cells were grown for 48 h; 5 × 10^5^ COLO357 and 1 × 10^4^ PANC-1 cells were plated in Matrigel^®^ invasion chambers (12-well format with 8 μM-sized pores; Corning, NY, USA) and grown overnight in the serum-free RPMI-1640 medium. SLURP1, nicotine, or both were added for another 24 h. After incubation, non-invading cells were removed from the upper surface of the membrane with cotton-tipped swabs. Subsequently, the membranes with the cells attached to the lower surface were fixed in PFA and stained with crystal violet. The digital images were taken, and the number of invaded cells was averaged from the four central fields digitally quantified using Bioanalyzer Software II (Agilent, Santa Clara, USA). In each experiment, the non-treated/non-transfected sample served as the100%-reference level to express other values as a percentage of the control. For statistical analysis and graphical presentation, the obtained values have been separated according to the CHRNA7-status into control (non-transfected and negative control siRNA-transfected samples) and CHRNA7-depletion (si#1 and si#3-tranfected samples) cohorts. The experiment was independently reproduced four times without transfection and two or three times with transfection. Obtained control vs. CHRNA7-depleted values (8 vs. 4 and 10 vs. 6 per each ligand-treated COLO357 and PANC-1 group, respectively) have been graphically presented and statistically analyzed as in scratch assay described above.

### MTT assay

For the MTT assay, non-transfected and siRNA-transfected 2.5–5 × 10^3^ PDAC cells were plated in triplicates into 96-well plates, starved overnight in serum-free medium, and then exposed to medium with 1% FCS and SLURP1, nicotine, or a combination of both. As controls, cells were left untreated at T_0 h_ and T_48 h_. MTT dye (3-(4,5-dimethylthiazol-2-yl)-2,5-diphenyltetrazolium bromide) was added during the last 4 h of the 48 h-long incubation; reduced MTT (formazan) was dissolved in isopropanol. Cell numbers were measured as absorbance at 570 nm, with growth effects calculated as a percentage of control = 100 × (T_48h sample_ - T_0h_)/(T_48h control_ - T_0h_). In each experiment, triplicates have been averaged; the non-treated/non-transfected sample served as the 100%-reference level to express other values as a percentage of the control. For statistical analysis and graphical presentation, the obtained values have been separated according to the CHRNA7-status into control (non-transfected and negative control siRNA-transfected samples) and CHRNA7-depletion cohorts (si#1, si#2, and si#3-tranfected samples). The experiment was independently reproduced nine (COLO357) and seven (PANC-1) times without transfection and three times with transfection. Obtained 13–15 control and 9 CHRNA7-depleted values per each ligand-treated group have been graphically presented and statistically analyzed as in scratch assay described above.

### Ca^2+^ influx assay

To determine changes in the intracellular Ca^2+^ concentration, 2 × 10^4^ PDAC cells were plated onto 96-well black microplates with clear bottoms and grown overnight. After treatment with SLURP1, nicotine, the specific CHRNA7 antagonist MLA, or the combinations, cells were loaded with FluoForte^®^ (FluoForte^®^ Calcium Assay Kit; Enzo^®^ Life Science, Farmingdale, NY, USA) which had been dissolved in Hank’s buffer with a dye efflux inhibitor. The kinetic measurement of fluorescence was performed using a Synergy^TM^ HTX Multi-Mode microplate reader (BioTek, Winooski, VT, USA).

### TransAM^®^ Transcription Factor EIA for NF-kB

To assess activation of the NF-kB pathway, 5 × 10^5^ PDAC cells were grown in complete medium in 6-well plates overnight, treated for 15 min with SLURP1, nicotine, a combination of both, or were left untreated. Nuclear extraction was performed using a nuclear extraction kit (Active Motif^®^, Rixensart, Belgium), and 10 μg of nuclear extract (Biuret-assay) was used to determine the amount of translocated NF-kB using the TransAM^®^ NFkB Family EIA Kit (Active Motif^®^). Ligands activity was determined as a log2-transformed treatment/control ratio (fold change).

### Statistical analyses

Statistical analyses were performed using commercially available software GraphPad Prism v5 (GraphPad Software, La Jolla, CA, USA) and IBM SPSS Statistics v22 (IBM Corp, Armank, NY, USA). Distribution of the experimental values was tested using D’Agostino and Pearson ‘omnibus K2’ normality test. All *in vitro* experiments were set in duplicates or quadruplicates, and repeated three to eight times. Technical duplicates were used to calculate a single mean value. The data obtained for non-transfected and negative siRNA set-transfected cells were treated as two independent measurements and were entered into the control cohort; three values for siRNA-treated cells/experiment were entered into the CHRNA7-depletion cohort. The non-transfected/non-treated sample was set as a 100%-reference (control) to normalize other values in each experiment. The graphs summarizing all *in vitro* data depict the percentage of control ± SEM. The differences between groups were explored using one-way ANOVA (multiple groups comparison) and Welch’s *t*-test (two groups comparison), and applied as indicated in the main text and figures’ legends. The graphs summarizing analyses of the clinical specimens (sera and tissues) depict median and interquartile range (IQR), the table list mean ± SEM. Frequencies of the CHRNA7 or SLURP1-positive specimens were compared with the Fisher’s exact-test. The relationships between an explanatory variable (serum SLURP1 or pancreatic CHRNA7 expression) and the outcome (the clinic-pathological parameters such as pT stage, pN stage, M stage, grade of differentiation/G and smoking status) were explored using a univariable binary logistic regression method. The prognostic relevance of the SLURP1 or CHRNA7 level was established in the context of clinico-pathological parameters using the multivariable Cox regression model; ^high^ and ^low^ groups were created using a publicly available Cut-off Finder Tool [84]. The impact of SLURP1 and CHRNA7 levels on survival was estimated using the Kaplan-Meier method and log-rank test. The significance level was set at 0.05 and indicated as ^*^ for *p* < 0.05, ^**^ for *p* < 0.01, and ^***^ for *p* < 0.001.

## Abbreviations

CHRNA7: cholinergic nicotinic receptor α7
CI: confidence interval
CP: chronic pancreatitis
EGF: epidermal growth factor
EIA: enzyme linked immunosorbent assay
FACS: Flow cytometry
HR: hazard ratio
MLA: methyllycaconitine
MS: median survival
MTT: 3-(4,5-dimethylthiazol-2-yl)-2
NF-kB: nuclear factor kappa B
NNK: nicotine-derived nitrosamine ketone
NNN: N-nitrosonornicotine
qRT-PCR: quantitative real time polymerase chain reaction
RPS6: ribosomal protein S6
RPS6K: ribosomal protein S6 kinase
PDAC: pancreatic ductal adenocarcinoma
SLURP1: secreted Ly-6/uPAR related protein 1
VEGF: vascular endothelial growth factor
